# Transcriptome analysis reveals key genes associated with root-lesion nematode *Pratylenchus thornei* resistance in chickpea

**DOI:** 10.1038/s41598-021-96906-3

**Published:** 2021-09-01

**Authors:** Sonal Channale, Danamma Kalavikatte, John P. Thompson, Himabindu Kudapa, Prasad Bajaj, Rajeev K. Varshney, Rebecca S. Zwart, Mahendar Thudi

**Affiliations:** 1 Centre for Crop Health, Institute for Life Sciences and the Environment, University of Southern Queensland (USQ), Toowoomba, Australia; 2 Center of Excellence in Genomics and Systems Biology (CEGSB), International Crops Research Institute for the Semi-Arid Tropics (ICRISAT), Hyderabad, India

**Keywords:** Plant stress responses, Plant biotechnology, Agricultural genetics, Biotechnology, Plant sciences

## Abstract

The root-lesion nematode, *Pratylenchus thornei,* is one of the major plant-parasitic nematode species causing significant yield losses in chickpea (*Cicer arietinum*). In order to identify the underlying mechanisms of resistance to *P. thornei,* the transcriptomes of control and inoculated roots of three chickpea genotypes viz. D05253 > F3TMWR2AB001 (resistant advanced breeding line), PBA HatTrick (moderately resistant cultivar), and Kyabra (susceptible cultivar) were studied at 20 and 50 days post inoculation using the RNA-seq approach. On analyzing the 633.3 million reads generated, 962 differentially expressed genes (DEGs) were identified. Comparative analysis revealed that the majority of DEGs upregulated in the resistant genotype were downregulated in the moderately resistant and susceptible genotypes. Transcription factor families WRKY and bZIP were uniquely expressed in the resistant genotype. The genes Cysteine-rich receptor-like protein kinase 10, Protein lifeguard-like, Protein detoxification, Bidirectional sugar transporter Sugars Will Eventually be Exported Transporters1 (SWEET1), and Subtilisin-like protease were found to play cross-functional roles in the resistant chickpea genotype against *P. thornei*. The identified candidate genes for resistance to *P. thornei* in chickpea can be explored further to develop markers and accelerate the introgression of *P. thornei* resistance into elite chickpea cultivars.

## Introduction

Chickpea (*Cicer arietinum*) is known to be the oldest cultivated legume, first domesticated around 10,000 years ago^1^. Presently, chickpea is grown in more than 55 countries, in diverse climatic conditions from tropical, through sub-tropical to temperate regions. Among plant-parasitic nematodes, *Pratylenchus thornei* is one of the most economically important root-lesion nematodes (RLN), widely distributed in chickpea growing areas throughout the world^2^. Like other *Pratylenchus* spp., *P. thornei,* are migratory endoparasites that penetrate plant root tissue with the help of a stylet and migrate intracellularly to withdraw nutrients from the cytoplasm of the root cortex cells. Yield losses in chickpea due to *P. thornei* in the two largest chickpea producing countries of the world have been estimated to be 25–30% in India^3^ and up to 25% in Australia^4^. Damage caused by RLN is easily misdiagnosed owing to indistinct above-ground symptoms. The reduced functionality of the host plant roots due to the damage caused by RLN feeding and reproducing inside the root cells results in similar symptoms to nutrient deficiencies, water stress, and soil-borne fungal pathogens viz., stunting, wilting, chlorotic leaves, reduced number of flower and pods, reduced yield and patchiness in the field^2^. The life-cycle of *Pratylenchus* lasts from 3 to 9 weeks depending on the species and conditions such as temperature, humidity, host and soil types^5^. *P. thornei* reproduces by parthenogenesis and the life-cycle starts with eggs that undergo differentiation to form the first stage juvenile (J1) within the egg and then the J1 moulting to J2 within the egg, before emerging on hatching. Through the subsequent life-cycle there are two more juvenile stages, J3 and J4, before the final moult into the fully developed adult.

Most chickpea cultivars are susceptible to *P. thornei*, allowing populations of the nematode to increase during crop growth and leaving high populations in the soil to attack other susceptible crops grown in rotation. The most sustainable approach currently used to manage RLN is crop rotation by growing non-host crops and resistant cultivars^6^. However, due to the broad host range of RLN, growing non-host crops may provide limited economical options for farmers. Thus, breeding resistant chickpea cultivars is a key strategy to manage RLN^7^. However, the domestication process has unintentionally led to the omission of important traits, leading to the narrow genetic base of chickpea^1^ and limited sources of high levels of *P. thornei* resistance in chickpea cultivars^7^.

In the case of cereals like wheat (*Triticum aestivum*), candidate genes responsible for resistance to *P. thornei* have been reported to encode key enzymes involved in the phenylpropanoid biosynthetic pathway, nucleotide-binding site-leucine-rich repeat (NBS-LRR) proteins and protein kinases^8^. In addition, recent metabolic profiling of another *P. thornei* resistant wheat genotype revealed abundance of flavonoids, fatty acid and lipids, alkaloids, tannins, nucleotides, steroid glycosides and terpenoids involved in defense signaling and pathways^9^. In the case of legumes like alfalfa (*Medicago sativa*), induction of the phenylpropanoid pathway and accumulation of secondary metabolites play critical roles in resistance to *P. penetrans*^10^.

The tremendous progress made in chickpea genomics during the last decade has provided significant insights into genome architecture^11^ and genome-wide variations^12,13^. These studies have enhanced our understanding of the genetic basis of several abiotic^14^ and biotic stresses^15^ and accelerated the development of climate resilient chickpea genotypes^15,16^. Stress responsive genes have been reported in chickpea using transcriptome analysis or RNA sequencing both for abiotic stresses as well as biotic stresses^17–19^. In addition, a global gene expression atlas revealing spatio-temporal changes during growth and development of chickpea has been developed^20^. Transcriptome studies can provide insights into candidate resistance genes and decipher the mechanisms of resistance in chickpea-*P. thornei* pathosystems, which are currently unknown.

In this study, we report candidate genes and pathways associated with *P. thornei* resistance in chickpea by utilizing a comparative transcriptomics approach. Understanding the mechanisms of resistance to *P. thornei* in chickpea will provide important insights for the design of novel, efficient and effective control strategies. Development of diagnostic molecular markers for key resistance genes will accelerate the introgression of *P. thornei* resistance into elite chickpea cultivars in breeding programs.

## Results

### Microscopy evaluation of *P. thornei* infection

**Figure 1:**
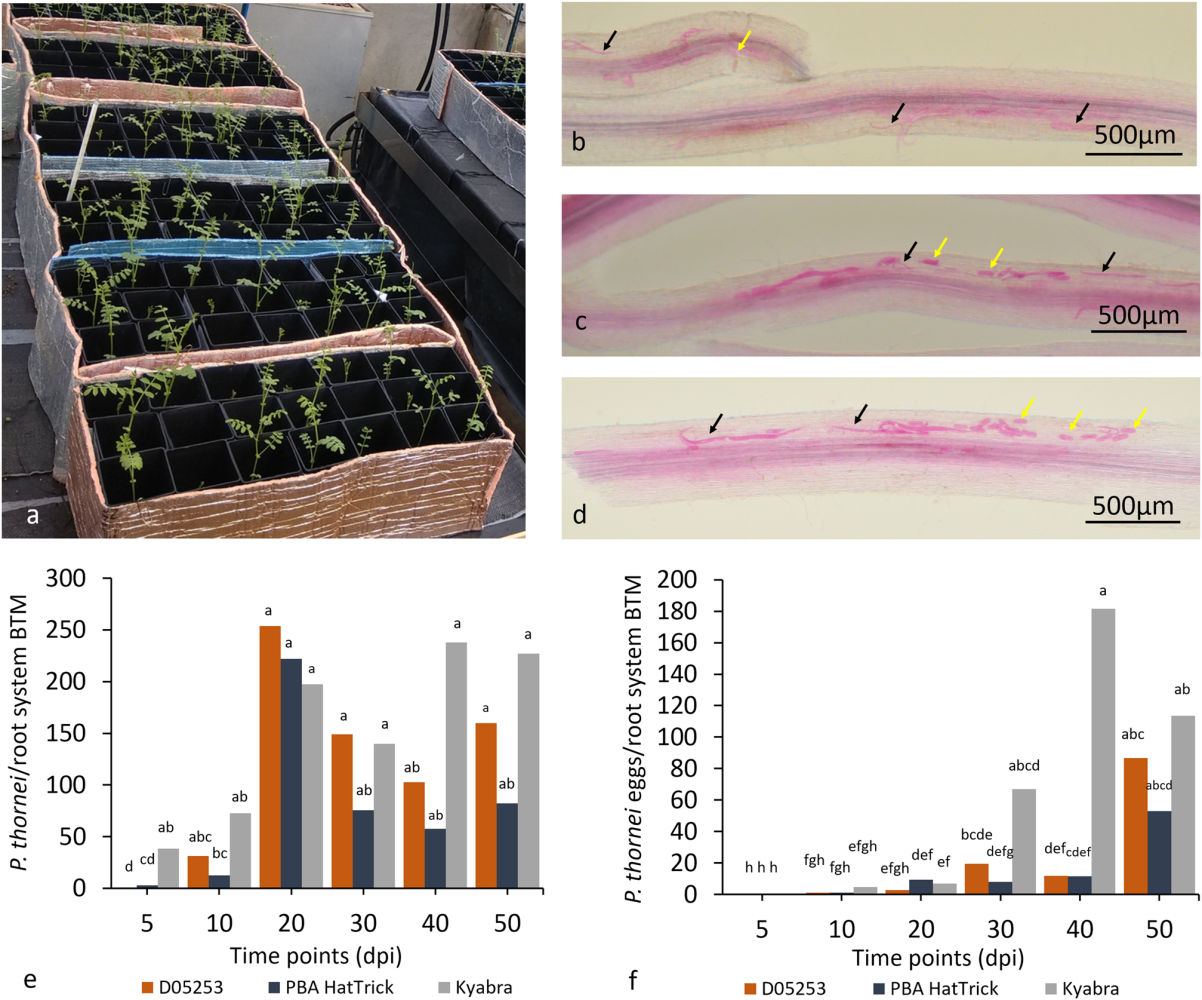
Phenotypic evaluation of *Pratylenchus thornei* infection of three chickpea genotypes D05253 (D), PBA HatTrick (H) and Kyabra (K) (**a**) Chickpea plants 5 days post inoculation (dpi) growing in the glasshouse. Roots stained with acid fuchsin at 50 dpi for chickpea genotypes (**b**) D05253, (**c**) PBA HatTrick, and (**d**) Kyabra, showing nematodes (black arrows) and eggs (yellow arrows). Time course from 5 to 50 dpi showing (**e**) *Pratylenchus thornei*/root system back transformed means (BTM), and (**f**) *P. thornei* eggs/root system back transformed means. Letters indicate l.s.d (*P* = 0.05) for mean transformed values ln(*x* + 1).

We examined and monitored the progress of nematode infection in a glasshouse pot experiment at 10, 20, 30, 40 and 50 days post inoculation (dpi) in the roots of three chickpea genotypes, namely, D05253 > F3TMWR2AB001 (resistant), PBA HatTrick (moderately resistant) and Kyabra (susceptible) (Fig. 1a). For ease, D05253 > F3TMWR2AB001 hereafter will be referred to as D05253. These observations confirmed infection of chickpea genotypes with *P. thornei* at the time points analyzed in the transcriptomics study (Fig. 1b–d). *P. thornei* adults and juveniles were observed in the roots of PBA HatTrick and Kyabra at 5 dpi, whereas for D05253 they were first observed at 10 dpi (Fig. 1e). There was no significant increase in the number of *P. thornei* for Kyabra from 5 dpi onwards and for D05253 from 10 dpi onwards. For PBA HatTrick, *P. thornei* numbers increased significantly (*P* < 0.02) between 5 dpi and time points from 20 dpi onwards. At 10 dpi and 20 dpi, *P. thornei* were more concentrated surrounding the root-shoot junction in comparison to any other part of the root system. For all time points after 20 dpi, *P. thornei* were observed more frequently along the length of both the tap root and lateral roots of Kyabra compared with PBA HatTrick and D05253. Eggs were first observed in all three genotypes from 10 dpi onwards (Figs. 1f). The number of eggs increased significantly between earlier time points for Kyabra (20 dpi to 30 dpi) (*P* = 0.03), compared with later time points (40 dpi to 50 dpi) for D05253 (*P* = 0.05) and PBA HatTrick. Eggs were clustered in groups of 4–5 in Kyabra, while in PBA HatTrick and D05253 eggs were found both singly and in clusters. Browning of the root tissue was observed in all the three genotypes from 10 dpi onwards, however, it was more pronounced from 40 dpi onwards in D05253 than in PBA HatTrick and Kyabra and was often accompanied with single or clusters of nematodes in the nearby tissue.

### Transcriptome sequencing and assembly

RNA-sequencing using Illumina HiSeq2500 of two time points, 20 dpi and 50 dpi, for inoculated and control treatments of the three genotypes D05253, PBA HatTrick, and Kyabra (a total of 12 samples), generated 633.30 million reads. The reads with adapter contamination and low base quality were removed and adapter free, good quality reads were obtained using Trimmomatic. As a result, a total of 533.95 million (84.35%) high quality reads were obtained following which, 520.28 million reads (97.44%) were mapped onto the chickpea reference genome (Supplementary Table S1).

### Differential gene expression analysis

Principal component analysis (PCA) revealed a clear difference among control and inoculated samples of D05253 and PBA HatTrick at 50 dpi, and Kyabra at 20 dpi, indicating expression differences among genotypes and time points (Supplementary Fig. S1). A gene was considered to be differentially expressed if |log2 (fold change)|≥ 2 at *P-*value ≤ 0.05 (Supplementary Table S2). On comparing control and inoculated samples of the three genotypes, a total of 962 (of which 693 were unique) differentially expressed genes (DEGs) were obtained (Supplementary Fig. S2). D05253 at 20 dpi (D20dpi) had 1.5 and 6 times more upregulated DEGs than PBA HatTrick at 20 dpi (H20dpi) and Kyabra at 20 dpi (K20dpi) respectively, whereas K20dpi had 3.5 and 12 times more down regulated DEGs than D20dpi and H20dpi, respectively. D05253 at 50 dpi (D50dpi) had 6 times more upregulated DEGs than both PBA HatTrick at 50 dpi (H50dpi) and Kyabra at 50 dpi (K50dpi), whereas H50dpi had 4 and 8 times more downregulated DEGs than D50dpi and K50dpi, respectively. The number of upregulated DEGs increased 3.4 times from D20dpi to D50 dpi, while the number of downregulated DEGs remained similar. The number of upregulated DEGs remained similar from H20dpi to H50dpi, while the number of downregulated DEGs increased 11 times. The number of upregulated DEGs were low in K20dpi and K50dpi, whereas the number of downregulated DEGs decreased by 9 times from K20dpi to K50dpi. When the three genotypes were compared at the same time point, none of the DEGs were common at 20 dpi, whereas at 50 dpi, four DEGs were found to be common (Supplementary Fig. S3). These four DEGs were Senescence-specific cysteine protease SAG39-like genes, which were upregulated in D50dpi and K50dpi but were downregulated in H50dpi. The two chickpea genotypes with resistance to *P. thornei* had three common DEGs at 20 dpi. All three DEGs were downregulated in D20dpi, whereas two were downregulated in H20dpi and one, putative Stigma-specific protein Stig1 gene, was upregulated. When D50dpi and H50dpi were compared, 99 DEGs were in common with most showing contrasting expression patterns between the genotypes (i.e., upregulation in D50dpi and downregulation in H50dpi), while four DEGs, belonging to plant physiological processes, were upregulated in H50dpi and one DEG was downregulated in D50dpi (Amino acid transporter AVT1H). All DEGs with annotations are listed in Supplementary Table S3.

### Functional classification of differentially expressed genes

The DEGs were further functionally categorized by Gene Ontology (GO) and Kyoto Encyclopedia of Genes and Genomes (KEGG). Out of the 693 unique genes which were differentially expressed, 471 genes were annotated to the GO database under the three groups (i) cellular component (CC), (ii) biological process (BP), and (iii) molecular function (MF) (Supplementary Table S3). The DEGs upregulated and downregulated in each group were analyzed for different subcategories. In D20dpi, for upregulated DEGs in BP category, “proteolysis involved in cellular protein catabolic process” (4 out of 31) was found to be the dominant term, whereas “lignin biosynthetic process” (3 out of 29) was the dominant term for downregulated DEGs. In CC category, “integral component of membrane” (11 out of 33) was found to be the dominant term for upregulated DEGs whereas, for downregulated DEGs, “integral component of membrane” and “nucleus” (each 5 out of 25) were found the dominant terms. For MF category, “cysteine-type endopeptidase activity” (4 out of 38) was the dominant term for upregulated DEGs and “DNA binding” (3 out of 34) was the dominant term for downregulated DEGs. For D50dpi, “meristem maintenance” (7 out of 117) was the dominant term in BP category for upregulated DEGs and that of “transmembrane transport” was the dominant term for downregulated DEGs. In MF category, “hydrolase activity” and “protein serine/threonine kinase activity” (each 5 out of 125) were dominant terms for upregulated DEGs. For downregulated DEGs in MF category, subcategory “transmembrane transporter activity” (4 out of 32) was found to be dominant. When H20dpi, subcategories were analyzed for BP category, “response to wounding term” (3 out of 30) was the dominant term for upregulated DEGs, whereas none of the subcategories were dominant for downregulated DEGs. For CC category “integral component of membrane” (9 out of 27) was the dominant term for upregulated DEGs and none was dominant for downregulated DEGs. For MF category, “DNA binding” (2 out of 27) was found to be the dominant term for upregulated DEGs and none was dominant for downregulated DEGs. For H50dpi, in BP category, subcategories “glutathione metabolic process” and “rejection of self pollen” were dominant (each 2 out of 19) and that of “carbohydrate metabolic process” and “regulation of transcription, DNA-templated” (each 6 out of 99) were dominant for downregulated DEGs. In CC category, “extracellular region”, “integral component of membrane” and “nucleus” (each 3 out of 19) were found be dominant terms among upregulated DEGs whereas “integral component of membrane” (28 out of 87) was the dominant term for downregulated DEGs. In MF category, subcategories “glutathione transferase activity” (2 out of 27) was the dominant term for upregulated DEGs and “cysteine-type endopeptidase activity” (5 out of 105) was the dominant term for downregulated DEGs. In K20dpi BP category, no terms were dominant for upregulated DEGs, however, “transmembrane transport” and “regulation of transcription, DNA-templated” (each 5 out of 94) were the dominant terms for downregulated DEGs. In CC category, “integral component of membrane” was the dominant term for both upregulated (6 out of 7) and downregulated (27 out of 86) DEGs. None of the terms were dominant for MF category for upregulated DEGs, however, “transmembrane transporter activity” was the dominant term for downregulated DEGs. Lastly, for K50dpi, in BP category, “proteolysis involved in cellular protein catabolic process” (4 out of 18) was the dominant term for upregulated DEGs, and “regulation of transcription” (3 out of 13) was the dominant term for downregulated DEGs. For CC category, subcategory, “extracellular space” (4 out of 14) was the dominant term for upregulated DEGs and that of “cytoplasm” (2 out of 10) was dominant for downregulated DEGs. In MF category, subcategory “cysteine-type endopeptidase activity” (4 out of 14) was the dominant term for upregulated DEGs and “glutathione oxidoreductase activity” (2 out of 11) was the dominant subcategory for downregulated DEGs. The DEGs were categorized into GO classification terms (Supplementary Fig. S4). Overall, the largest categories were “transmembrane transport” for BP, “integral component of membrane” for CC, and “metal ion binding” for MF.

To gain further insight into the biological significance of the DEGs, GO enrichment analysis was performed (Supplementary Table S4). In BP category, “phenylpropanoid metabolic process” (*P* = 0.000142), “regulation of defense response” (*P* = 0.000450) and 10 other terms were significantly over-represented, whereas “organelle organization” (*P* = 4.52E−06), “peptide biosynthetic process” (*P* = 4.94E−06) and 33 other terms were significantly under-represented. In MF category, “oxidoreductase activity” (*P* = 6.24E−06) was significantly over-represented along with 15 other terms, whereas as “nucleic acid binding” (*P* = 2.29E−10) was significantly under-represented with 12 other terms. In CC category, “extracellular region” (*P* = 6.34E−12) was significantly over-represented along with four other terms, whereas “intracellular anatomical structure” (*P* = 1.16E−12) was significantly under-represented along with 17 other terms.

KEGG pathway analysis was performed to obtain an overall view of pathways involved in *P. thornei*-chickpea interactions (Supplementary Table S5). The highest number of enzymes belonged to pathways related to biosysnthesis of antibiotics (11), phenylpropanoid biosythesis (5), pentose and glucuronate interconversions (5) and starch and sucrose metabolism (5).

### Differentially expressed transcription factor families

The upregulated transcription factor (TF) families involved in plant immune response and accumulation of secondary metabolites were identified as basic helix-loop-helix (bHLH) proteins TF family (22), ERF (13), MYB-related (13), NAC (11), AP2 (5), WRKY (4) and bZIP (2) (Supplementary Fig. S5). Both bZIP and WRKY TF families were uniquely expressed in D05253. Also, MYB-related and AP2 TF families were predominanlty expressed in D05253 at 20 dpi and 50 dpi, absent from PBA HatTrick, and only one of each TF family was upregulated in Kyabra at 50 dpi.

### Validation of gene expression profiles by quantitative real time PCR analysis

**Figure 2:**
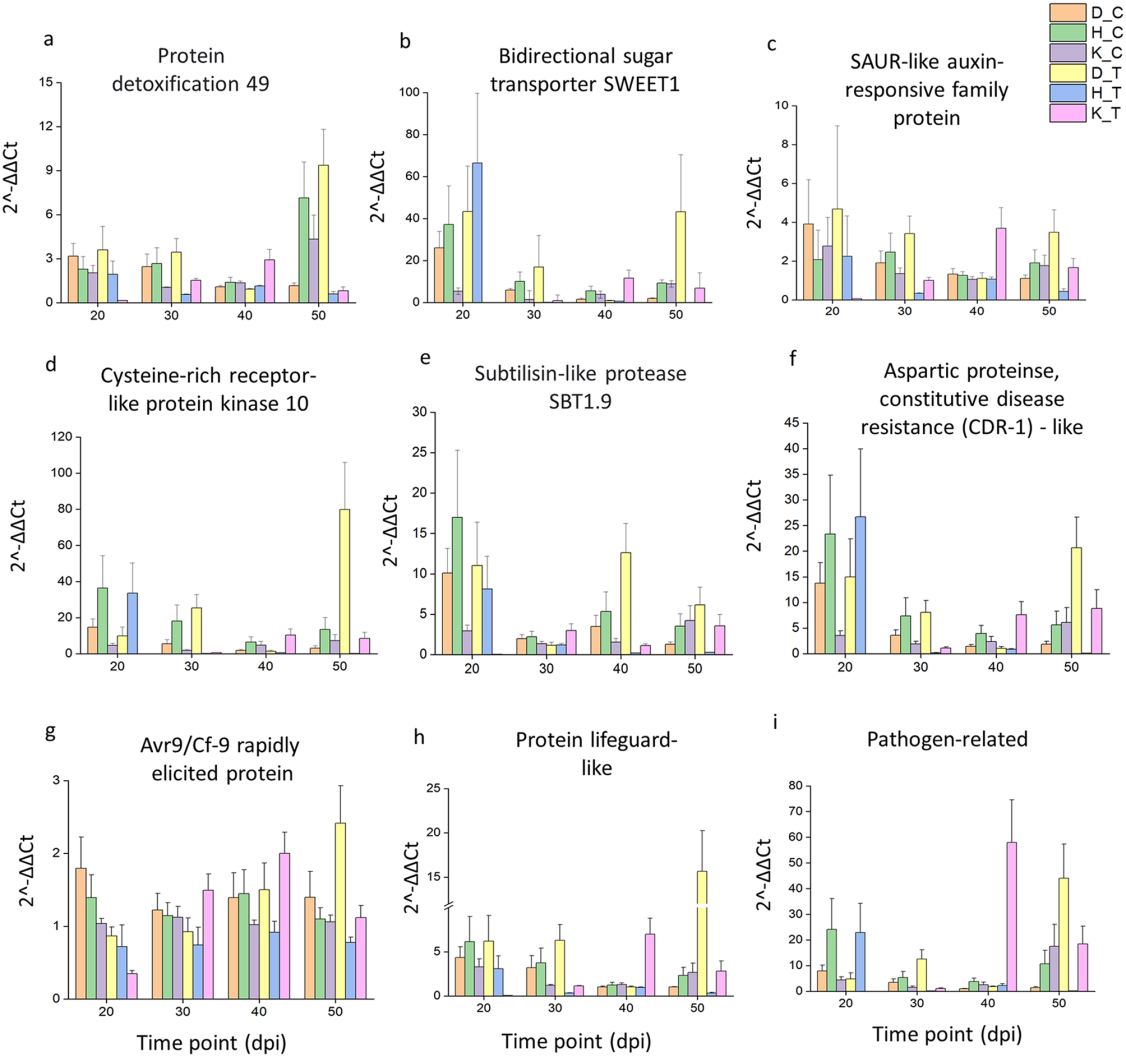
The qRT-PCR analysis of important genes across different time points in three genotypes. D_C, D05253 control; D_T, D05253 treated; H_C, PBA HatTrick control; H_T, PBA HatTrick treated; K_C, Kyabra control; K_T, Kyabra treated. Error bars represent standard deviation of the mean with four independent biological replicates. CRK-10 (Cysteine-rich receptor-like protein kinase 10); SAUR (Small auxin-upregulated RNA)-like auxin responsive family protein); Bidirectional sugar transporter SWEET1 (Sugars Will Eventually be Exported Transporters1). The graphs were generated using Origin (OriginLab Corp., Northampton, USA).

**Figure 3:**
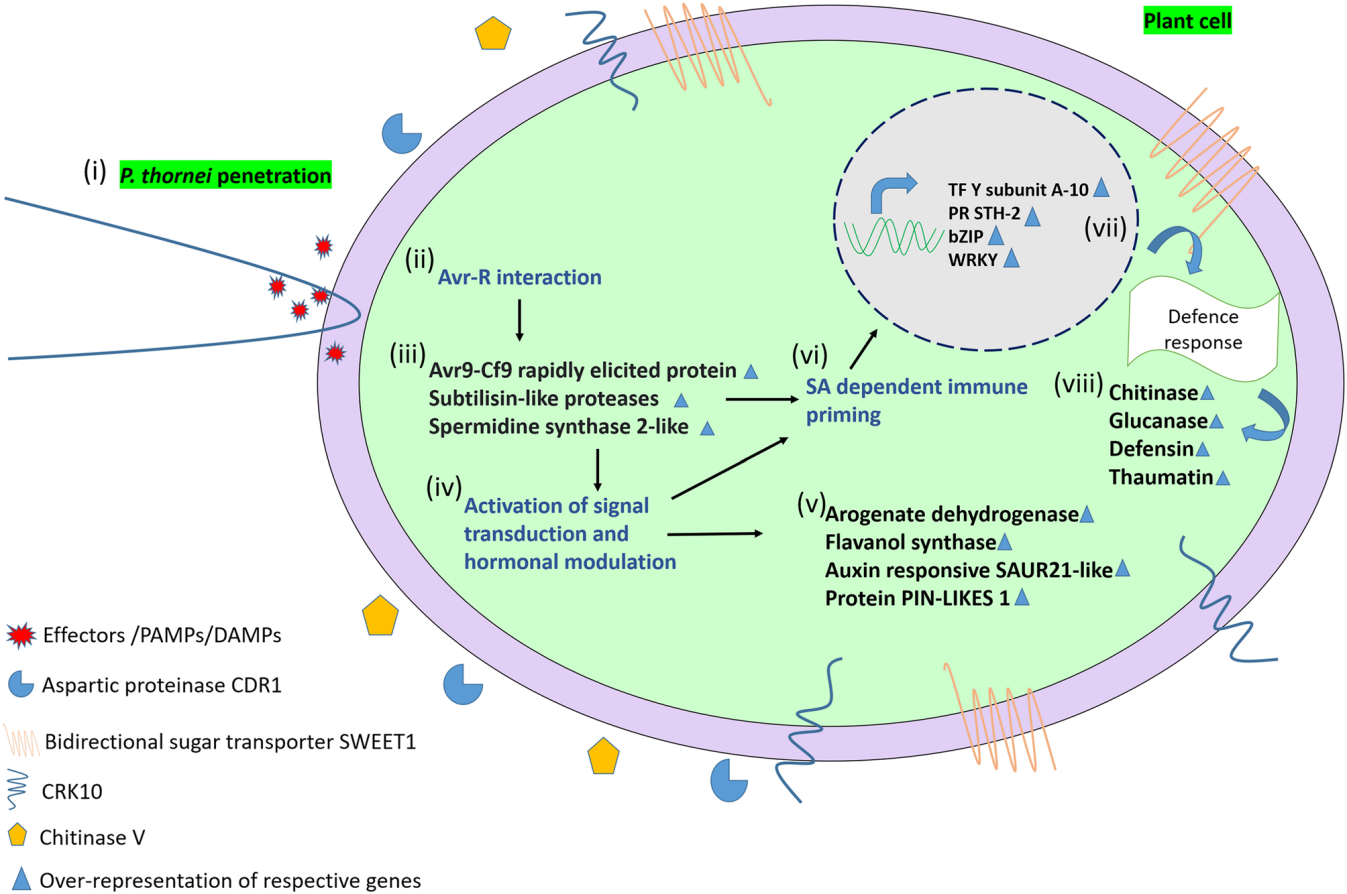
The cascade of events following *P. thornei* invasion in resistant chickpea genotype based on transcriptome data: (i) *P. thornei* penetration in chickpea root cells (ii) Avr-R interaction on *P. thornei* invasion which leads to (iii) Upregulation of Avr9-Cf9 rapidly elicited protein, Subtilisin-like proteases and Spermidine synthase 2-like (iv) Activation of signal transduction and hormonal regulation which results in (v) Upregulation of Arogenate dehydrogenase, Flavanol synthase, Auxin responsive SAUR21-like and Protein PIN-LIKES 1. Events taking place in (ii) and (iii) leads to (vi) Salicylic acid (SA) dependent immune priming leading to (vii) Transcriptional regulation of genes governing defence response (viii) Upregulation of Chitinase, Glucanase, Defensin and Thaumatin. Avr, Avirulence protein; R, Resistance protein; CDR1, Constitutive disease resistance 1; CRK10, cysteine-rich receptor-like protein kinase 10; Bidirectional sugar transporter SWEET1, Sugars Will Eventually be Exported Transporters1; PAMPs, pathogen‐associated molecular patterns; DAMP, damage associated molecular patterns; SAUR, Small Auxin Upregulated RNAs.

The accuracy of the RNA-seq data was verified by quantitative real time PCR (qRT-PCR) using 12 genes (Supplementary Table S6). The expression values of the RNA-seq and qRT-PCR results for each gene ranged from *r* = 0.78 to 0.99, with an experiment-wise correlation of* r* = 0.76 (*P* < 0.001), indicating that the qRT-PCR expression patterns were consistent with the results obtained by RNA-seq (Supplementary Fig. S6). Following qRT-PCR validation, nine DEGs, namely Protein detoxification 49, Bidirectional sugar transporter Sugars Will Eventually be Exported Transporters1 (SWEET1), Small Auxin Upregulated RNA (SAUR)-like auxin-responsive, Cysteine-rich receptor-like protein kinase 10 (CRK 10), Subtilisin-like protease SBT1.9, Aspartic proteinase constitutive disease resistance (CDR-1)-like, Avr9/Cf-9 rapidly elicited protein, Protein lifeguard-like and Pathogen-related genes, were evaluated at time points 20, 30, 40 and 50 dpi using qRT-PCR (Fig. 2). Protein detoxification 49 and Avr9/Cf-9 rapidly elicited protein were upregulated only at 50 dpi by eight and twofold differences respectively in D05253. Other DEGs namely Bidirectional sugar transporter SWEET1, CRK 10, Aspartic proteinase CDR-1-like, Protein lifeguard-like and Pathogen-related were upregulated intermittently ie; at 30 dpi and 50 dpi by ≥ twofold and > tenfold, respectively in D05253. (SAUR)-like auxin responsive gene was also upregulated at 30 and 50 dpi by four-and fivefold, respectively in D05253. Whereas, Subtilisin-like protease SBT1.9 was upregulated at 40 dpi and 50 dpi by four- and fivefold, respectively, in D05253. A model depicting the transcriptome responses to *P. thornei* involving various genes in the resistant genotype D05253 is shown in Fig. 3.

## Discussion

Understanding the molecular mechanisms and candidate genes involved in *P. thornei* resistance will be useful for designing stategies to overcome crop production losses. This study is the first comprehensive investigation into the molecular mechanisms of resistance to *P. thornei* in chickpea. Gene expression profiling in resistant and suceptible chickpea genotypes revealed differences in timing and gene expression levels in reponse to *P. thornei* infestation. Unique transcriptional patterns of defense-related genes were observed in the *P. thornei* resistant advanced breeding line D05253. Gene expression profiling showed an opposite trend for several important genes, with common genes upregulated in D05253 at 50 dpi, down regulated in PBA HatTrick at 50 dpi, as well as in Kyabra at 20 dpi. These genes were categorized as (i) defense responsive, (ii) stress responsive, (iii) genes involved in plant growth and development, and (iv) genes involved in secondary metabolite pathways.

The response of chickpea plants to infestation by *P. thornei* is characterized by the interplay of a cascade of signaling pathways. Plant defense mechanisms depend on the initial recognition of the pathogen^21^. Migrating and feeding RLN induce damage-associated molecular patterns (DAMPs) and also secrete effectors, proteolytic enzymes, and other proteins that may elicit pathogen-associated molecular patterns (PAMPs)^5^. The perception is carried out by a large family of cell membrane pattern-recognition receptors (PRRs) which are either receptor-like kinases (RLKs) or receptor-like proteins (RLPs)^22^. The Cysteine (C)-rich receptor-like kinases (CRK) subfamily, part of receptor-like protein kinases (RLKs), are plasma membrane proteins, which have a role in signal sensing and activating systemic acquired resistance (SAR)^23^. Upregulation of CRK10 in the resistant genotype D05253 inoculated with *P. thornei* was observed at 30 dpi and 50 dpi, while no differential expression of this gene was observed in PBA HatTrick and Kyabra, defining its possible role in perception of *P. thornei* invasion in the resistant chickpea genotype. RLKs have been reported to be candidate genes for *P. thornei* resistance in wheat^8^*.* Also, enhanced resistance from RLKs, i.e., CRKs, has been demonstrated in other host plant-pathogen systems. For example, overexpression of Arabidopsis CRK4, CRK6, and CRK36 enhanced the activation of early and late (PAMP)-triggered immunity (PTI) responses and enhanced resistance to the bacterial pathogen *Pseudomonas syringae* pv tomato^24^. CRK10 when overexpressed, resulted in enhanced resistance to *Xanthomonas oryzae* pv. *oyzae,* the causal agent of rice bacterial leaf blight^23^. Onset of SAR leads to activation of various PR proteins, which act as diagnostic markers for defense signaling^25^ In support of this observation, the different PR proteins upregulated only in D05253 were glucanase, defensin-like protein 1 and thaumatin-like protein.

Remarkably, Avr9/Cf-9 rapidly elicited protein gene was found to be upregulated only in the resistant chickpea genotype D05253 at 50 dpi, suggestive of a plausible resistance gene mediated defense system in the chickpea-*P. thornei* interaction. The Cf-9, resistance gene product in tomato acts like a receptor for the corresponding Avr9 protein in *Cladosporium fulvum* and results in a chain of reactions for activation of plant endogenous defense systems. These ACRE (Avr9/Cf-9 rapidly elicited) proteins are protein kinases, transcription factors and components of E3 ligase complexes, which play important roles in Cf-9 mediated resistance^26^.

Limiting nutrient access is one immune response that can be initiated when plants react to pathogens, or when pathogens develop strategies to access nutrients and suppress plant immunity^27^. The recent studies conducted on plant pathogen interactions have reported the importance of sugar transporters, which can alter the source-sink sugars to fulfil the energy demands of plant or pathogen^28^. Interestingly, Bidirectional sugar transporter SWEET1 (Sugars Will Eventually be Exported Transporters1) was upregulated at 30 dpi and 50 dpi in D05253, indicating the role of *P. thornei* infestation in modulation of sugar transporters. Overexpression of SWEET4 in *Vitis vinifera* hairy root was demonstrated to promote flavonoid biosynthesis and increased resistance against *Pythium irregulare*^29^*.* In addition to Bidirectional sugar transporter SWEET1, upregulation of Arogenate dehydrogenase, required for tyrosine biosynthesis, and which acts as a precursor for major classes of secondary metabolites^30^ was observed in D05253 at 50 dpi.

Plant proteases play an important role in defense responses against invading pathogens^31^. Constitutive disease resistance 1 (CDR1) is an apoplastic aspartic protease that induces local and systemic signaling in *Arabidopsis thaliana*. CDR1 was also found to be upregulated in D05253 and PBA HatTrick at 20 dpi. CDR1 thus, might have processed a cell surface protein that could be a component of the basal host defense complex, or alternatively, it may release an extracellular mobile peptide as an elicitor that activated host basal defense responses^32^ in *P. thornei* resistant chickpea genotypes. CDR1 was identified in *A. thaliana* to mediate peptide signalling systems and activate inducible resistance against virulent *Pseudomonas syringae*^33^*.*

Plants generally secrete chitinases as a defensive action upon fungal infection^34^. Chitin is the main component of the egg shell of nematodes^35^. Chitinases are known to induce premature hatching and could damage the cuticle of plant-parasitic nematodes during various life-stages^36^. The present study revealed the upregulation of Chitinase V in the resistant genotype, whereas for the moderately resistant and susceptible genotypes the expression level was considerably lower or downregulated. Similarly, increased chitinase activity and upregulation of transcript levels was also observed in resistant plants challenged with root-knot nematode *Meloidogyne* incognita^37^.

Plant subtilisin-like proteases (subtilases) are serine proteases, involved in a range of biological functions such as development, cell wall modification, processing of peptide growth factors, and in responses to biotic and abiotic stress^38^. In the present study, Subtilisin-like protease SBT1.9 was constitutively expressed in D05253 and PBA HatTrick at 20 dpi, while induced in D05253 only at later time points of 40 dpi and 50 dpi, suggesting that SBT1.9 might be involved in enhancing the immune response in D05253. *Arabidopsis* subtilase (SBT3.3) has been reported to enhance an innate immune response, and undergo an auto-induction mechanism that promotes chromatin remodeling and activates salicylic acid (SA) dependent immune priming of defense genes^39^.

Under-representation of Small Auxin Upregulated RNA (SAUR)-like auxin-responsive family protein and other auxin responsive genes were observed in PBA HatTrick and Kyabra, while upregulation at 30 dpi and 50 dpi was observed for D05253. SAUR family is one of the important gene families that are involved in auxin signalling-regulated plant growth and development. Furthermore, SAUR family is also known to be involved in various biotic and abiotic stress responses, suggestive of cross-talk between different processes^40^. In particular, it has been generally observed that, auxin, auxin related genes and SA act antagonistically^41^. However, upregulation of auxin related DEGs and SA responsive PR DEGs in the present study suggests possible synergistic interactions wherein the plant is trying to cope with the *P. thornei* stress by fine-tuning the growth and developmental processes and defense responses.

Abscisic acid (ABA) is an important hormone that regulates plant developmental processes and can function as a positive or negative regulator of plant defense depending on the specific plant-pathogen interaction. Negative crosstalk of ABA and SA has been reported to be necessary for resistance in plan-pathogen interactions^41^. In the present study, ABA responsive DEGs were not detected in any of the genotypes, which could be partly due to the negative regulation of ABA in the presence of SA signalling pathway.

Transcription factors play important roles in regulating and integrating the various immune responses as a result of biotic or abiotic stress. WRKY and bZIP TFs were uniquely expressed in the resistant genotype D05253. Studies on plant-nematode interactions have demonstrated the role of WRKY transcription factors in defense mechanisms. WRKY TFs have been reported to contribute to resistance against root-knot nematodes^42^ and cyst nematodes. Several WRKY genes analyzed respond to pathogen attack and to the signaling molecule SA^43^. bZIP TFs play vital roles in plant innate immunity due to their ability to regulate genes associated with PAMP-triggered immunity, effector-triggered immunity, and hormonal signaling networks^44^. In addition to the DEGs mentioned above, other DEGs were found to be important in stress responses and defense response against *P. thornei* and were upregulated in D05253 only, namely Pathogenesis-related thaumatin family protein, Pathogenesis-related protein STH-2-like, Leucine-rich repeat receptor-like serine/threonine-protein kinase At1g17230 isoform X, Universal stress family protein, RING-H2 finger protein ATL66. Spermidine synthase 2, enzyme involved in synthesis of polyamine spermine was also upregulated only in D05253.

Overall, this study illustrates expression of several DEGs common to biotic and nematode stress, along with different transcription factors regulating them. *P. thornei* with the virtue of its biotrophic nature, was able to successfully overcome immune responses in PBA HatTrick and Kyabra. However, the resistant genotype D05253, with successful perception of NAMPs/PAMPs, followed by activation of SAR was able to mount a strong immune response against *P. thornei*. As a result, numerous genes participated in the cross-talk in different defense pathways to provide resistance against *P. thornei* in chickpea*,* suggesting *P. thornei* resistance in chickpea is polygenic, as in wheat^45^ and thereby also making it more durable than that of monogenic resistance. Molecular markers derived from these candidate genes thus can be incorporated in breeding programs for marker assisted selection.

## Methods

### Plant material and inoculum

Three *C. arietinum* genotypes ranging in resistance and susceptibility to *P. thornei* viz., an advanced breeding line D05253 (resistant), cv. PBA HatTrick (moderately-resistant) and cv. Kyabra (susceptible) were used in this study. The *P. thornei* resistance of D05253 is derived from the wild relative *C. recticulatum* ILWC140 introgressed into adpated Australian chickpea germplasm^46,47^. PBA HatTrick and Kyabra are high yielding commercially relevant cultivars widely grown in Australia. The strain of *P. thornei* used in this study was originally isolated from a field site in Formartin, Queensland, Australia (latitude 27.46°S, longitude 151.43°E) and cultured for inoculum^48^. The percent of adults and juveniles in the inoculum was 27% and 73%, respectively. All experiments on these materials were performed in accordance with relevant institutional, national and international guidelines and legislation.

### Plant growth conditions

Glasshouse pot experiments were conducted at the Leslie Research Facility (Toowoomba, Australia) on benches fitted with a bottom watering system^49^. The chickpea seeds were surface sterilized with 2% sodium hypochlorite for 2 min and washed with sterile Milli-Q water eight times, then pre-germinated for 48 h on moist filter paper at room-temperature. Germinated seed was planted in pots (70 mm square base × 150 mm high) containing 264 g (equivalent to 80% total capacity) of sterile 3:7 soil-sand mixture. The soil was a field collected vertosol. For planting, a small hole was made in soil-sand mix, enough for the seed radical to be inserted, with the radical facing downwards and the seed was covered by the sand-soil mixture. After 4 days, inoculated treatments 3,300 *P. thornei* (equivalent to 10,000 nematodes/kg oven-dry soil-sand mixture) in 10 mL of aqueous suspension were applied around the plant base, while control plants were mock inoculated. Pots were then capped with the remaining (20%) soil-sand mixture (equivalent to 66 g).

### Experimental design

Two glasshouse experiments were conducted in parallel, with both experiments inoculated with the same batch of *P. thornei* inoculum and grown on adjacent benches, namely: (i) Microscopy evaluation of *P. thornei* infection and (ii) Transcriptome experiment. Treatments in the first experiment were used to confirm nematode penetration in the chickpea plants and monitor the progress of *P. thornei* infection over time using microscopy, while the treatments in the second experiment were used for the transcriptome studies. The microscopy evaluation experiment consisted of the three genotypes (D05253, PBA HatTrick and Kyabra) inoculated with *P. thornei*, by six harvest time points (5, 10, 20, 30, 40 and 50 dpi) arranged in a randomized complete block design with three independent biological replications. The transcriptome experiment consisted of a factorial split-plot design with the three chickpea genotypes, two nematode treatments (*P. thornei* inoculated and control), and the six time points for root tissue harvest (as in the first experiment) and six independent biological replications. Within replicates, inoculated and control treatments were randomly assigned as main plots and separated by a distance of 15 cm to avoid nematode contamination of control pots, while genotype × time point treatments were randomly assigned within each sub-plot. Experimental designs were generated using agricolae package in R^50^.

### Microscopy evaluation of *P. thornei* infection

#### Sample collection

Whole root systems from each treatment in the first experiment were harvested at the specified time points by separating the roots from the sand-soil medium and washing with tap water. The plant roots were stained with 0.1% acid fuchsin^51^. Stained nematodes and eggs were observed inside the roots using a stereo microscope (Olympus SZH, Japan) and images obtained on Olympus MVX10 with a 0.63X objective (MV PLAPO) and digital camera at × 60 (DP74, Olympus, Macquarie Park, Australia) using cellSens Olympus Dimension software version 1.17.

#### Statistical analysis

Nematode and egg count data were transformed by ln(*x* + 1), where *x* = number of *P. thornei*/root system or number of *P. thornei* eggs/root system, to conform to a normal distribution with homogeneity of variance required for analysis of variance (ANOVA), as assessed by graphical diagnostics of the residual plots. Transformed data were analyzed by ANOVA and least significant differences (l.s.d.) of the predicted means were calculated using the aov and lsd functions of the PredictMeans package^52^ in R. The data was then back transformed by exponentiation.

### Transcriptome experiment

#### Sample collection

The roots were harvested at the different time points as per the experimental design by removing the whole plant from the pot. The roots were separated from the medium, gently cleaned with 0.1% diethyl pyrocarbonate (DEPC) treated water, blotted dry on filter paper, excised at the root-shoot junction, flash frozen in liquid nitrogen and stored at -80 °C until RNA extraction.

#### RNA extraction, illumina sequencing

Roots were finely crushed in liquid nitrogen using a pre-chilled mortar and pestle. Total RNA was extracted from 800 mg of finely crushed roots using Spectrum Plant Total RNA kit (Sigma-Aldrich, Code# STRN250-1KT, Missouri, USA), followed by on-column DNase digestion (Sigma-Aldrich, Code# DNASE70-SET, Missouri, USA) as per the manufacturer's instructions. The quantity and quality of the RNA were determined using Qubit RNA estimation kit (Life Technologies, Code# Q32855, Eugene, Oregon, USA) and Agilent 2100 Bioanalyzer (Agilent Technologies, Santa Clara, USA). For Illumina sequencing, two time points, 20 dpi and 50 dpi were selected, based on microscopy evaluation of the infection process and consideration that the life cycle of *Pratylenchus* is completed in approximately 45 days^53^. To ensure the integrity of RNA for sequencing, biological replicates for each treatment had a RNA integrity number (RIN) ≥ 8. Three biological replicates were pooled, resulting in 12 samples for sequencing (three genotypes × two nematode treatments (inoculated and mock inoculated control)) × two time points (20 dpi and 50 dpi).

#### Reference-based mapping and assembly and data quality control

The raw dataset was checked for its quality using FASTQC version 0.11.8 (http://www.bioinformatics.babraham.ac.uk/projects/fastqc/) and filtered using Trimmomatic-0.39^5^ for trimming and cleaning any adapters or overrepresented sequences present in the dataset. The high-quality filtered reads were aligned to the CDC-frontier chickpea reference genome sequence^11^ using HISAT2^55^ set to default parameters. The aligned transcripts were assembled using StringTie-v2.1.0^56^ to obtain assembled transcripts for each sample separately that estimated the levels of expression of each gene and isoform. The assembled transcripts were merged using the StringTie-merge function that merges all the assemblies obtained in order to create a consistent set of transcripts across all samples for the two time points. The StringTie further provides additional read-count data for each transcript, which are further required by DESeq (differential expression analysis for sequence count data)^57^. Differential expression analysis was performed using the DESeq R/Bioconductor package parameter method ‘blind’ for non-replicate data. Variance stabilized data was used to perform Principal Component Analysis (PCA) through ClustVis^58^.

#### Identification of differentially expressed genes (DEGs), Gene Ontology (GO) and pathway analysis

To obtain significant DEGs, a minimum difference of a twofold change with *P*-value ≤ 0.05 was used as a threshold. The basemean values (a measurement of normalized numbers of mapped reads) from the RNA-seq data set were log_2_-transformed and the differential genes that exhibited log_2_ transformed values ≤ -2 were considered to be downregulated genes and those ≥  + 2 were considered to be upregulated genes. The sequences of these DEGs were further extracted and aligned using standalone blast + version 2.7.1^59^ against nr database taxon viridiplantae for functional annotations. The Gene Ontology (GO) annotation, GO enrichment and Kyoto Encyclopedia of Genes and Genomes (KEGG) pathway analysis was performed using Blast2GO v5^60^. Furthermore, the DEGs were searched against the Plant transcription factor database (PlantTFDB 4.0) for identification of transcription factor encoding genes. The specific TF families involved in plant immune response and accumulation of secondary metabolites to combat stress form a large portion of the TF repertoire in plants^61^ and were analyzed for the DEGs across all the genotypes and time points.

#### Quantitative PCR analysis and expression across different time points

Quantitative real time PCR was performed for 12 DEGs identified from *in-silico* data analysis to confirm RNA-Seq data. The 12 DEGs were selected covering a range of expression levels based on log_2_FC (≥ 2 and ≤ 5 as low expression level, > 5 and ≤ 8 as moderate expression level, > 8 as high expression level) in D05253 and functional categorization based on GO analysis. Primers were designed with Primer-BLAST^62^ and are listed in the Supplementary Table S6. The total RNA was reverse-transcribed using High-Capacity cDNA Reverse Transcription Kit (Applied Biosystems, Code# 4374966, Carlsbad, CA, USA), following the manufacturer’s protocol. The cDNA synthesis was confirmed using housekeeping gene Glyceraldehyde 3-phosphate dehydrogenase (GAPDH) followed by dilution and normalization for qRT-PCR analysis. The qRT-PCR was performed using QuantStudio3 (Applied Biosystems, Thermo Fisher Scientific, Carlsbad, CA, USA) with SYBR® PremixExTaq™ II (Tli RNaseH Plus) (Takara Bio, Code # RR820W, TaKaRa, Dalian, China) for four independent biological replicates and three technical replicates. Briefly, the 10-μL reaction mixture contained 5 μL enzyme mix with 0.25X ROX II, 3.6 μL H_2_O, 0.2 μL forward primer (10 mM), 0.2 μL reverse primer (10 mM), and 1 μL sample of cDNA. The expression values were calculated using the 2^−ΔΔCt^ method^63^. Mean values and standard deviations were obtained from four biological replicates and three technical replications. The correlation coefficient (r) between qRT-PCR and RNA-seq data was calculated. Following qRT-PCR validation at time points 20 dpi and 50 dpi, *P. thornei* responsive genes were assessed for the three genotypes, inoculated and control, at time points 20, 30, 40 and 50 dpi, to observe the trend of over-representation or under-representation of genes over time.

## Supplementary Information

Supplementary Information 1.

Supplementary Information 2.

## Data Availability

The datasets generated during the current study are available in the NCBI Sequence Read Archive (SRA) under the BioProject Accession No. PRJNA715023.
