# Publisher Correction: Transcriptome analysis reveals key genes associated with root-lesion nematode *Pratylenchus thornei* resistance in chickpea

**DOI:** 10.1038/s41598-022-08495-4

**Published:** 2022-03-11

**Authors:** Sonal Channale, Danamma Kalavikatte, John P. Thompson, Himabindu Kudapa, Prasad Bajaj, Rajeev K. Varshney, Rebecca S. Zwart, Mahendar Thudi

**Affiliations:** 1 Centre for Crop Health, Institute for Life Sciences and the Environment, University of Southern Queensland (USQ), Toowoomba, Australia; 2 Center of Excellence in Genomics and Systems Biology (CEGSB), International Crops Research Institute for the Semi-Arid Tropics (ICRISAT), Hyderabad, India

Correction to: *Scientific Reports* 10.1038/s41598-021-96906-3, published online 01 September 2021

The Funding section in the original version of this Article was omitted. The Funding section now reads:

“Authors are thankful to Science Engineering Research Board (SERB; Grant ID CRG/2018/003056) for funding this work”.

The original Article has been corrected.

