# Supplementary material for: Transcriptome analysis reveals key genes associated with root-lesion nematode Pratylenchus thornei resistance in chickpea
Source: Sci Rep. 2021 Sep 1;11:17491. doi: 10.1038/s41598-021-96906-3 (PMC8410808; doi:10.1038/s41598-021-96906-3)
Supplement: Supplementary file 1 — Supplementary Information 1. [file 41598_2021_96906_MOESM1_ESM.pdf]

Supplementary Figures

RNA-Seq analysis reveals key genes associated with root-lesion nematode (*Pratylenchus thornei*) resistance in chickpea

Sonal Channale[1], Danamma Kalavikatte[2], John P. Thompson[1], Himabindu Kudapa[2], Prasad Bajaj[2], Rajeev K. Varshney[2], Rebecca S. Zwart[1]*, Mahendar Thudi[1,2] *

[1]Centre for Crop Health, Institute for Life Sciences and the Environment, University of Southern Queensland, Toowoomba, Australia
[2]Center of Excellence in Genomics & Systems Biology (CEGSB), International Crops Research Institute for the Semi-Arid Tropics (ICRISAT), Hyderabad, India

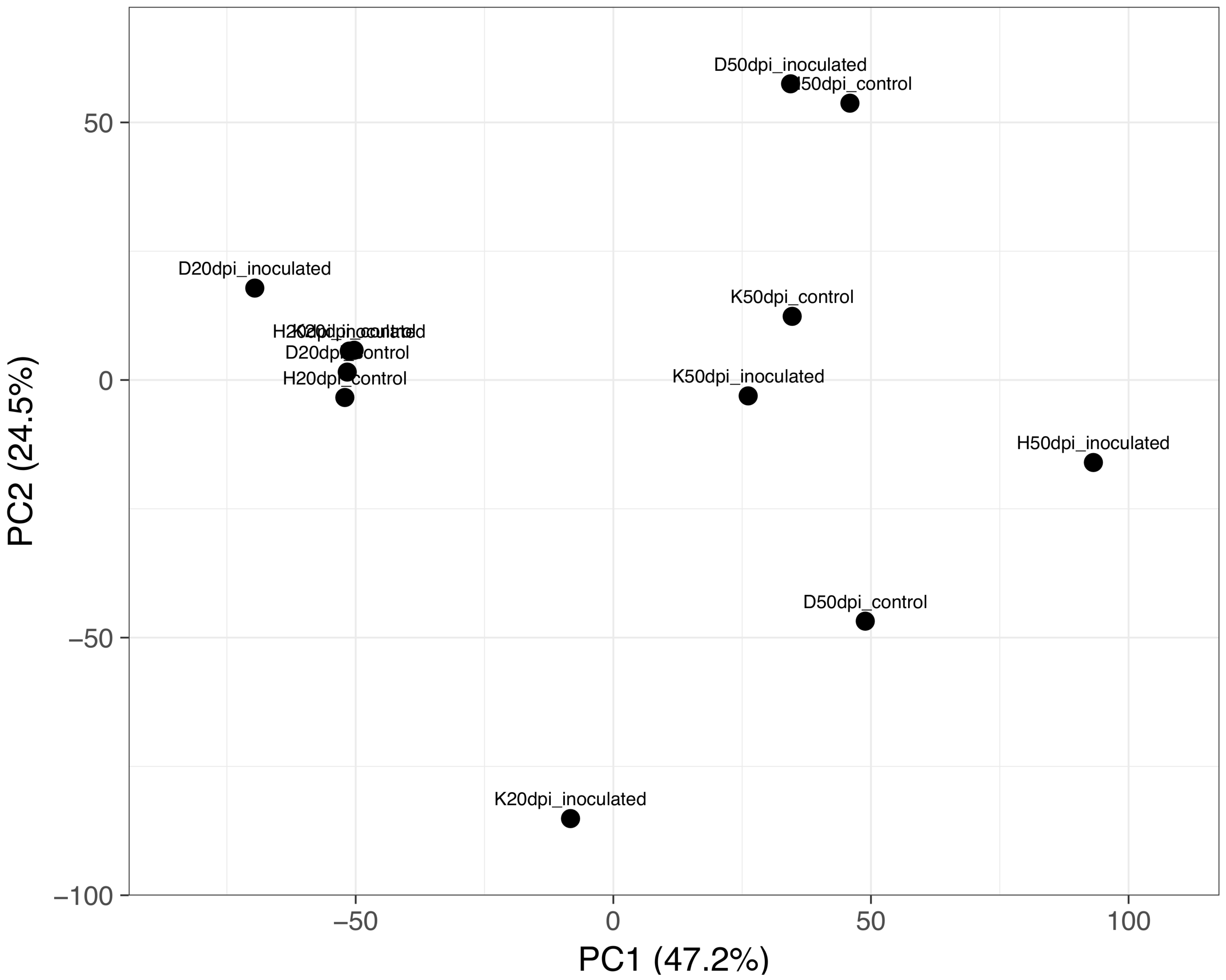

Supplementary Figure S1: Principal component analysis (PCA) reveals a clear difference among control and inoculated samples of D05253 >F3TMWR2AB001 (D) at 50 days post inoculation (dpi) with *Pratylenchus thornei*, PBA HatTrick (H) at 50 dpi and Kyabra (K) at 20 dpi. The PCA plot was generated using ClustVis tool (https://biit.cs.ut.ee/clustvis/).

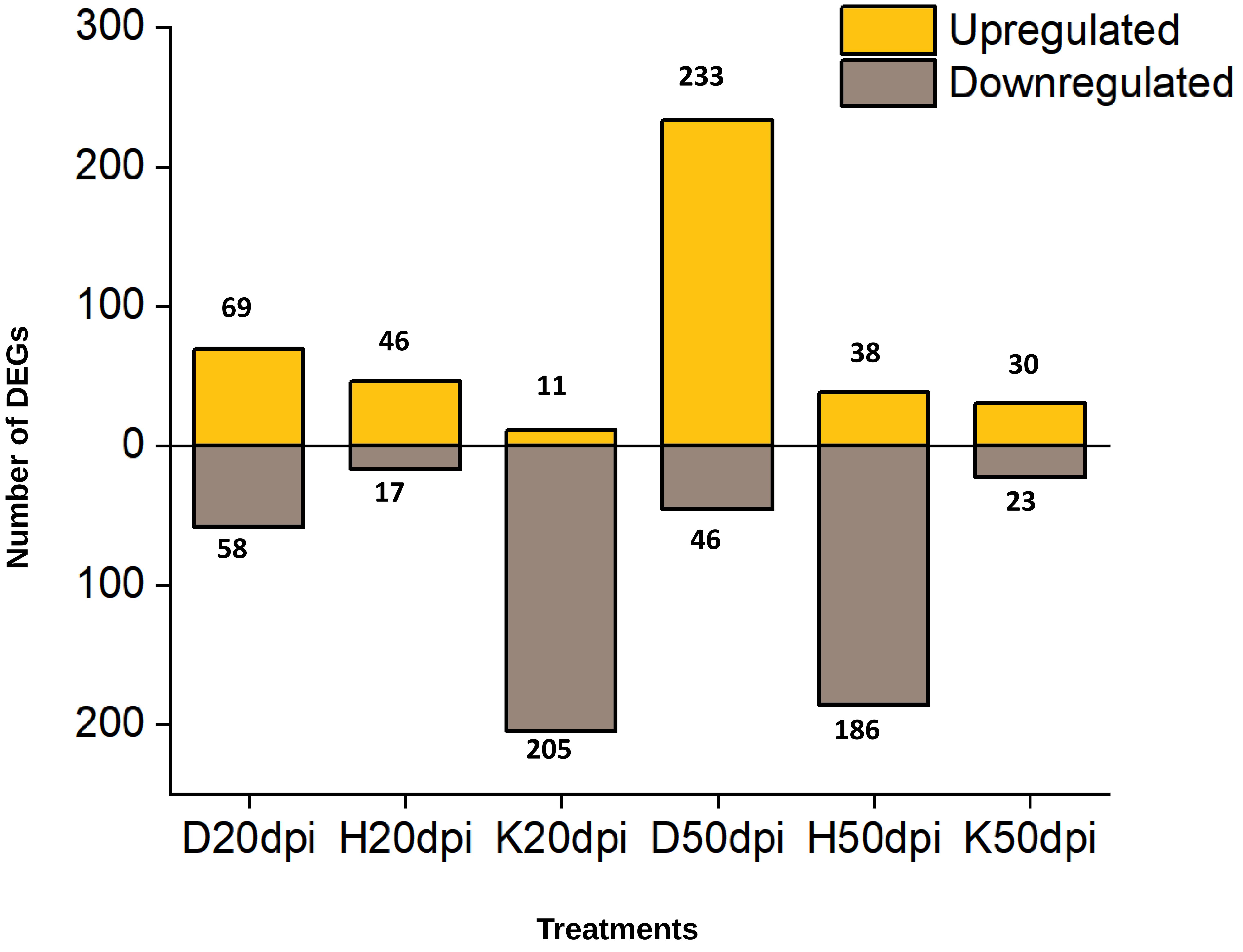

Supplementary Figure S2: Number of significant differentially expressed genes (DEGs) (log_2 fold change ≥2 or ≤2 and a P-value ≤ 0.05) in three chickpea genotypes D05253 >F3TMWR2AB001 (D); PBA HatTrick (H); Kyabra (K) at 20 and 50 days post inoculation (dpi) with *Pratylenchus thornei*. Bars above the x-axis represent the number of upregulated genes relative to the control plants at each time point, while bars below the line represent the number of genes downregulated. The graph was generated using Origin (OriginLab Corp., Northampton, USA).

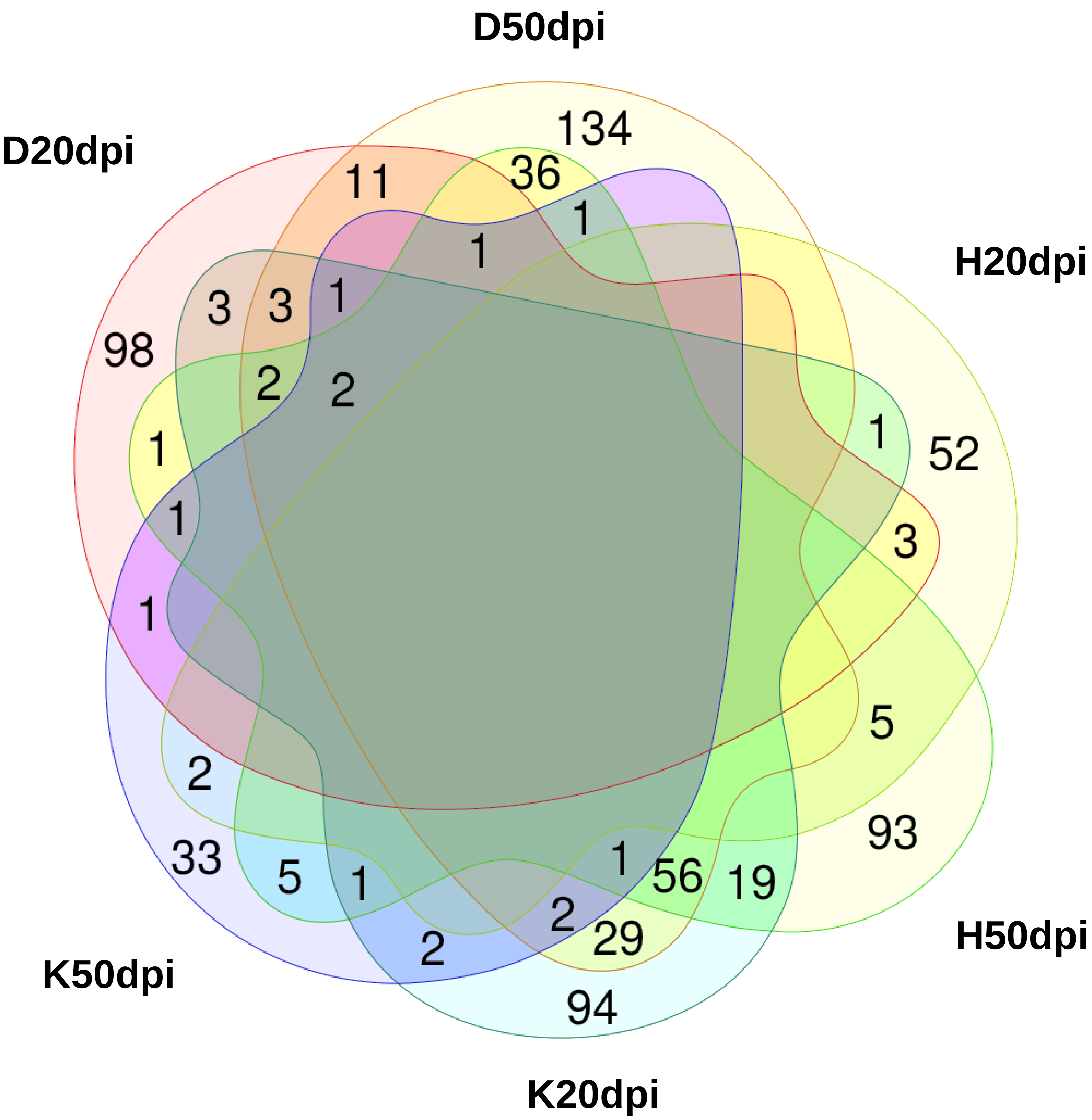

Supplementary Figure S3: Venn diagram showing the number of differentially expressed genes (DEGs) in common between the three chickpea genotypes, D05253>F3TMWR2AB001 (D), PBA HatTrick (H); Kyabra (K), and two time points, 20 and 50 days post inoculation (dpi) with *Pratylenchus thornei*. The Venn diagram was generated using VennDiagram R package version 1.6.20.

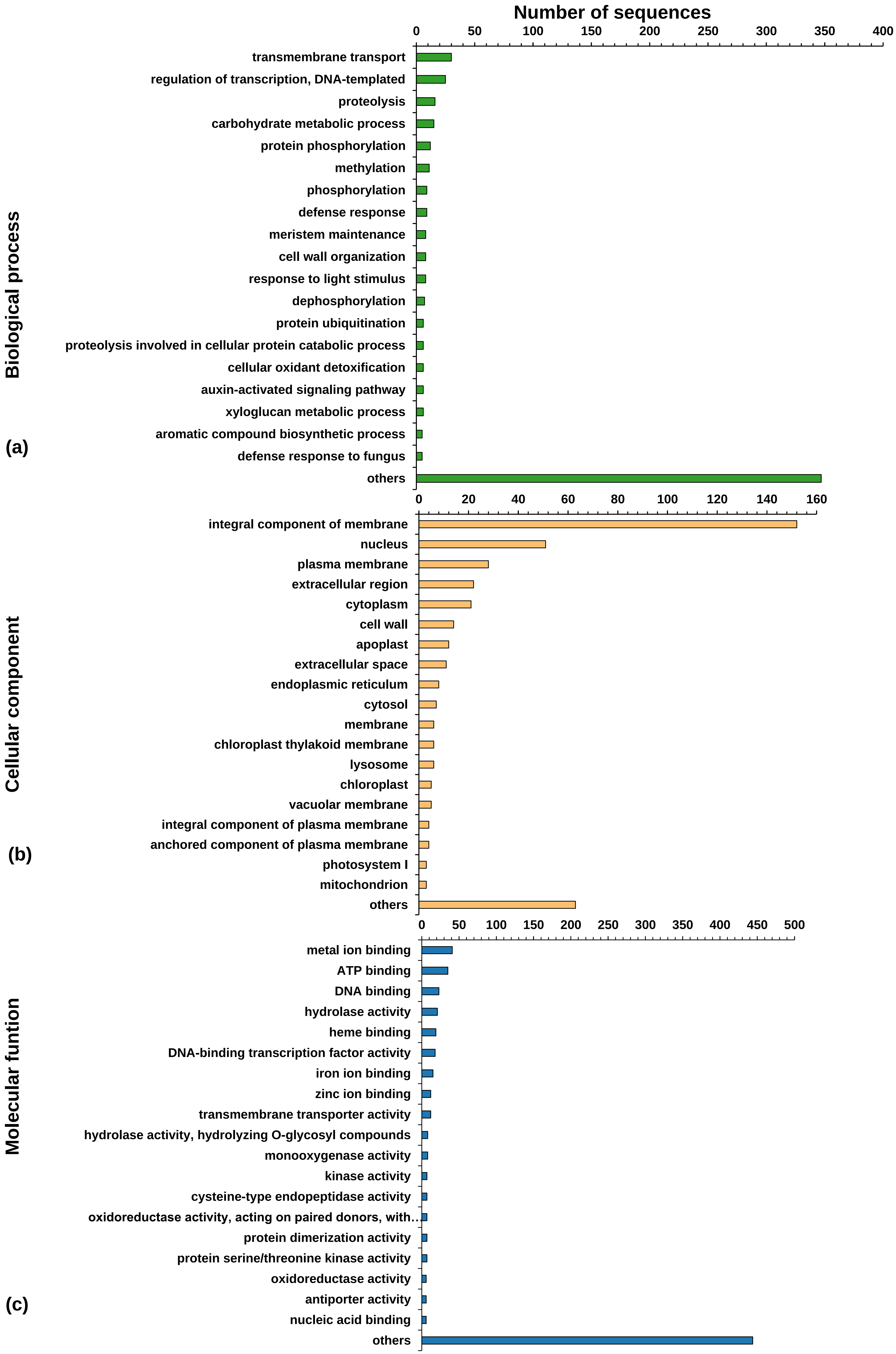

Supplementary Figure S4: Gene Ontology (GO) analysis using top 20 GO classification terms of 962 differentially expressed genes in chickpea genotypes D05253>F3TMWR2AB001, PBA HatTrick and Kyabra at time points 20 and 50 days post inoculation (dpi) with *Pratylenchus thornei*, categorized into (**a**) biological process (BP) (**b**) molecular function (MP), and (**c**) cellular component (CC). Blast2GO version 5.2 was used for generating the graphs.

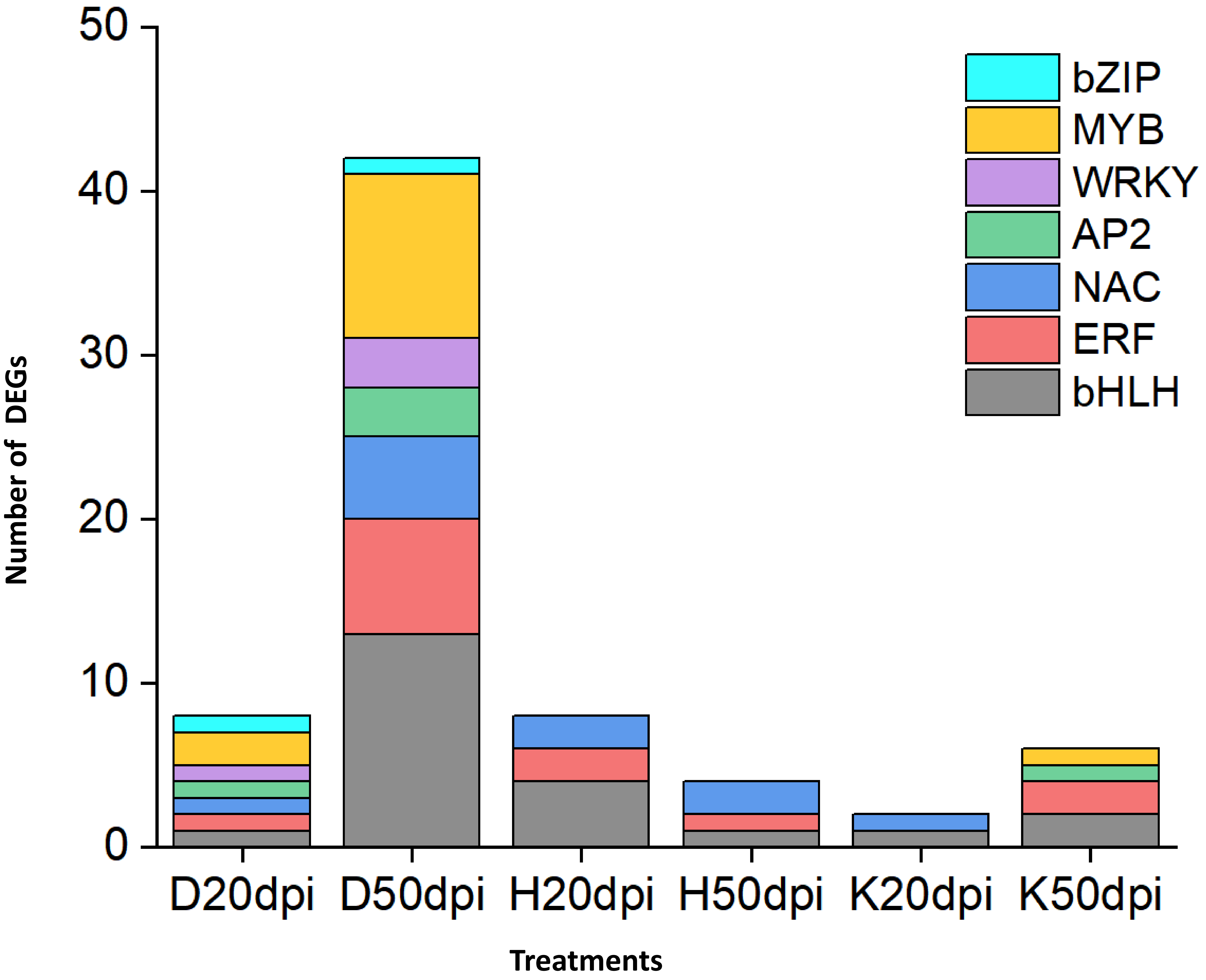

Supplementary Figure S5: Transcription factor families upregulated across chickpea genotypes D05253>F3TMWR2AB001 (D), PBA HatTrick (H) and Kyabra (K) at time points 20 and 50 days post inoculation (dpi) with *Pratylenchus thornei*. The graph was generated using Origin (OriginLab Corp., Northampton, USA).

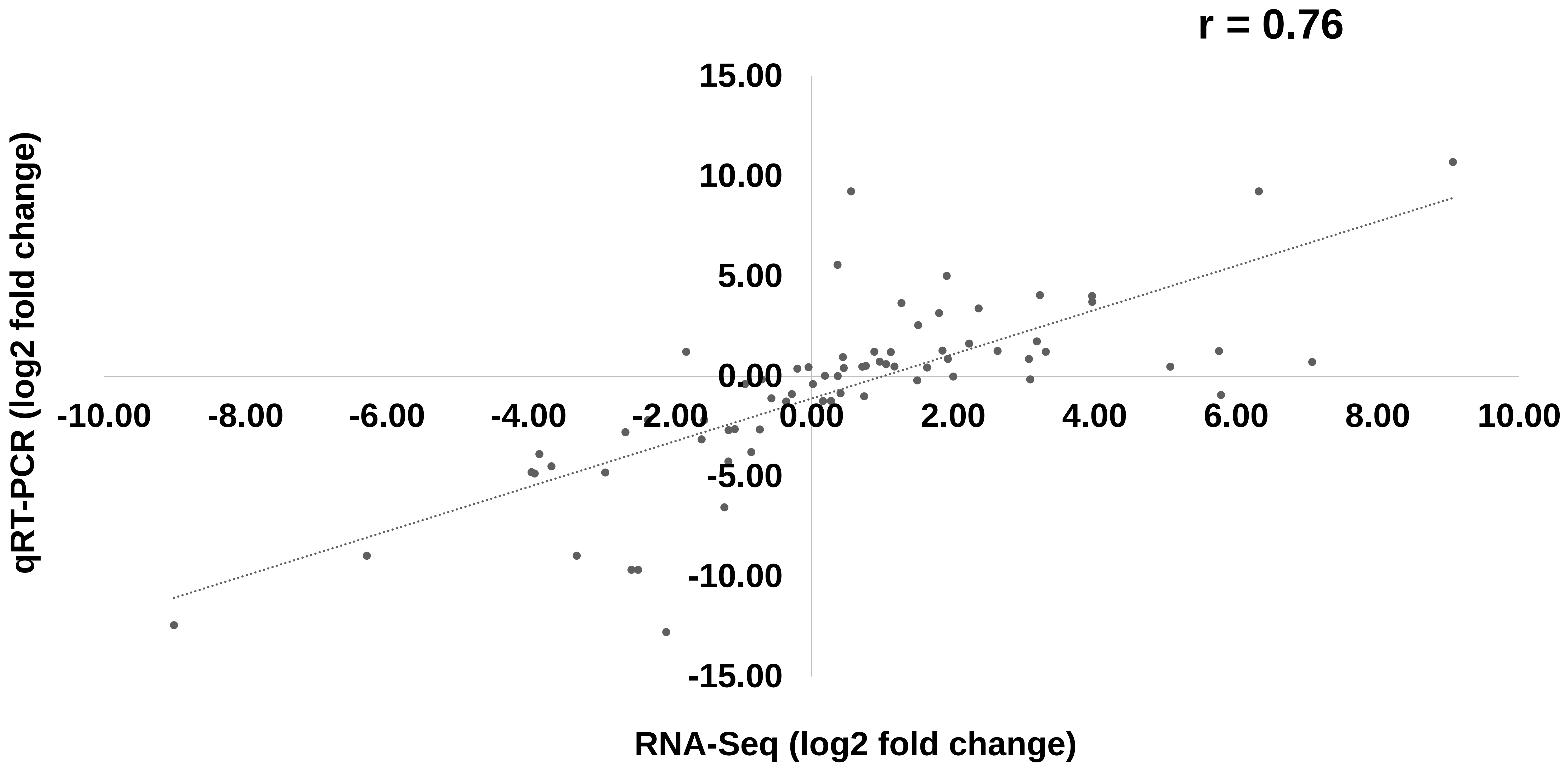

Supplementary Figure S6: Correlation of expression level (log$_2$ fold change) of nine selected genes between the two approaches, RNA-Seq and q-RT PCR, for three chickpea genotypes, D05253>F3TMWR2AB001, PBA HatTrick and Kyabra at time points 20 and 50 days post inoculation (dpi) with *Pratylenchus thornei* (n=54).
